# A Test of Concept Study of At-Home, Self-Administered HIV Testing With Web-Based Peer Counseling Via Video Chat for Men Who Have Sex With Men

**DOI:** 10.2196/publichealth.6377

**Published:** 2016-12-14

**Authors:** Jessica L Maksut, Lisa A Eaton, Elizabeth J Siembida, Daniel D Driffin, Robert Baldwin

**Affiliations:** ^1^ Department of Human Development and Family Studies University of Connecticut Storrs Mansfield, CT United States; ^2^ Institute for Collaboration on Health, Intervention, and Policy University of Connecticut Storrs Mansfield, CT United States

**Keywords:** HIV prevention, barriers to HIV testing, HIV stigma, MSM, Web-based, Internet, video chat

## Abstract

**Background:**

Men who have sex with men (MSM), particularly MSM who identify as African-American or Black (BMSM), are the sociodemographic group that is most heavily burdened by the human immunodeficiency virus (HIV) epidemic in the United States. To meet national HIV testing goals, there must be a greater emphasis on novel ways to promote and deliver HIV testing to MSM. Obstacles to standard, clinic-based HIV testing include concerns about stigmatization or recognition at in-person testing sites, as well as the inability to access a testing site due to logistical barriers.

**Objective:**

This study examined the feasibility of self-administered, at-home HIV testing with Web-based peer counseling to MSM by using an interactive video chatting method. The aims of this study were to (1) determine whether individuals would participate in at-home HIV testing with video chat–based test counseling with a peer counselor, (2) address logistical barriers to HIV testing that individuals who report risk for HIV transmission may experience, and (3) reduce anticipated HIV stigma, a primary psychosocial barrier to HIV testing. ** **

**Methods:**

In response to the gap in HIV testing, a pilot study was developed and implemented via mailed, at-home HIV test kits, accompanied by HIV counseling with a peer counselor via video chat. A total of 20 MSM were enrolled in this test of concept study, 80% of whom identified as BMSM.

**Results:**

All participants reported that at-home HIV testing with a peer counseling via video chat was a satisfying experience. The majority of participants (13/18, 72%) said they would prefer for their next HIV testing and counseling experience to be at home with Web-based video chat peer counseling, as opposed to testing in an office or clinic setting. Participants were less likely to report logistical and emotional barriers to HIV testing at the 6-week and 3-month follow-ups.

**Conclusions:**

The results of this study suggest that self-administered HIV testing with Web-based peer counseling is feasible and that MSM find it to be a satisfactory means by which they can access their test results. This study can serve as a general guideline for future, larger-scale studies of Web-based HIV test counseling for MSM.

## Introduction

### Background

Men who have sex with men (MSM), and in particular, MSM who identify as African-American or Black (BMSM), historically have been, and continue to be, the group most heavily impacted by the human immunodeficiency virus (HIV) epidemic in the United States [1,2]. While MSM comprise approximately 2% of the US population, 67% of all new HIV diagnoses in 2014 occurred among men who identify as gay, bisexual, or same gender loving [3]. President Obama’s Updated National HIV/AIDS Strategy calls for expanded efforts to prevent HIV by using a combination of effective, evidence-based approaches to improve uptake of HIV prevention and treatment strategies [4]. While there have been advances in improving the adoption of these strategies among MSM [5-8], there continues to be an urgent need for further work that addresses the issue of access to comprehensive HIV care services, including HIV testing, for this population.

For MSM, the most commonly cited barriers to clinic- or office-based HIV testing include logistical concerns (eg, inconvenience of a testing site’s hours of operation [9]) and psychosocial issues (eg, fear of discrimination, disclosure of one’s sexual identity [10]). Pertinent social determinants of MSM’s health, including perceived sexual orientation–based stigmatization by medical professionals, and associated mistrust of the medical community, act as barriers to accessing HIV care, including routine HIV testing and counseling. Indeed, there is evidence that fear of stigmatization may negatively impact patients’ health-related quality of life, adherence to treatment regimens, and HIV risk behaviors [11]. In light of the facts that (1) HIV testing is a critical entry point to engagement in HIV care, and (2) multiple barriers discourage MSM from routinely HIV testing, it is worth exploring novel, nonstandard approaches to offering HIV testing services to this population.

In an effort to expand upon current testing approaches, alternative venue HIV prevention outreach efforts that target MSM have emerged. These approaches include behavioral education programs focused on HIV testing uptake, mobile HIV testing in public and semipublic places (eg, parks, streets, community spaces), and linking HIV testing with lesbian, gay, bisexual, and transgender–identified services (eg, community centers) [12]. While beneficial, it is of concern that these approaches may not reach MSM who are not open about their sexual orientation and/or behavior, those who are younger, and those who report less education [13], as well as MSM who would prefer to test in the privacy of their own home [14]. Alternative routes to HIV testing and counseling that are easy to access, offer relative anonymity, can be conducted at home, and offer support by a peer educator may be particularly appealing for MSM.

There exists evidence for the benefits of incorporating Internet delivery as a modality for increasing HIV testing uptake. A recent systematic review of Web-, mobile phone–, and social media–based interventions to address the HIV continuum of care by Muessig et al [15] found that 5 Web-based HIV testing interventions had been published, and that an additional 8 were currently in development. Included in the review was a social media–based HIV testing intervention, Project Hope, which randomly assigned peer leaders to deliver either HIV information and promotion of free home-based HIV tests (ie, intervention) or general health information (ie, control) via social media [16]. Young et al [16] found that the intervention group participants were more likely to request home-based HIV testing kits, and take and return test kits than control participants. A number of additional Web-based studies [17-20] with similar formats to those mentioned above (Web-, mobile phone–, and social media–based) aimed at promoting HIV testing exist, but according to Schnall et al [21], they vary greatly with respect to study quality, and the majority, unlike Project Hope, aim to increase rates of clinic-based HIV testing. One such example is a Web-based HIV testing intervention by Bauermeister and colleagues [22], which offered tailored, motivational content around HIV/sexually transmitted infections (STIs) testing as well as a testing locator to MSM. While Bauermeister et al [22] found that those who received the intervention (compared with those who received the testing locator only) were more likely to visit an HIV/STI testing clinic, it is possible that some participants still may have not tested due to barriers to clinic-based testing. Like Bauermeister et al, Zou et al [23] found that active (eg, instant messaging, chat rooms, mobile phones, email) promotion methods were more effective at promoting HIV testing than passive (eg, posters) methods. Taken together, previous Web-based studies of HIV testing point to the usefulness of the Internet’s reach, as well as the importance of active methods to promote HIV testing; however, most studies were structured around motivating participants to access clinic-based testing rather than bringing testing directly to individuals by mailing test kits.

Now that there are at-home HIV test kits available, more should be done to understand how the Internet can augment and improve the experience of HIV testing at home. According to Katz et al [24], who conducted a study on the acceptability and ease of use of at-home HIV test kits among MSM, 96% of MSM found the test kits very easy to use, while the remaining participants found them somewhat easy to use. The majority of participants in Katz et al’s study [24] requested additional kits after the first. Choko et al [25] found in their study on the uptake and accuracy of at-home, oral HIV test kits that accuracy was good, though approximately 10% of participants made minor procedural errors and 10% reported wanting or needing supervisory support. Based on the previously mentioned review of prior Web-based HIV testing interventions, as well as the evidence of the accuracy, acceptability, and ease of use of at-home testing, offering Web-based HIV counseling to accompany at-home HIV testing experiences appears to be a relatively untapped area for intervention development.

### Study Objectives

The current study examined the feasibility of self-administered, at-home HIV testing with Web-based peer counseling to MSM by using an interactive video chatting method (eg, Skype, Google Hangouts, FaceTime). The primary aim was to conduct a test of concept study to determine whether individuals would participate in at-home HIV testing with video chat–based test counseling with a peer counselor. A secondary aim was to gather preliminary evidence that this testing modality could (1) address logistical barriers to HIV testing that individuals who report risk for HIV transmission may experience, and (2) reduce anticipated HIV stigma, a primary psychosocial barrier to HIV testing. Pre- and posttesting assessments were used to evaluate changes in variables of interest, including barriers to HIV testing and anticipated HIV stigma.

## Methods

### Participants

Recruitment occurred through word of mouth, phone call-ins, and Web-based advertisements on dating websites for gay and bisexual men. Recruitment targeted BMSM in particular, though MSM who were not Black/African-American but had heard about the study via word of mouth and expressed interest were still invited to participate. Participants were recruited over a 3-month period from January to March 2015. To be eligible, participants reported having condomless anal sex with a man in the past 6 months; having an HIV negative or unknown status; being at least 18 years of age; having access to a computer, tablet, mobile phone, or other device with video chatting capabilities and Internet/mobile service; and agreed to receive an HIV test kit via mail. Twenty men in the Atlanta, GA metropolitan area provided informed consent and were enrolled. Of those, 18 participants completed the 6-week and 3-month follow-up assessments. Participants were provided monetary incentives for completing survey assessments, specifically US $35 for completing the baseline appointment and US $25 for each follow-up appointment. All study procedures were approved by the University of Connecticut institutional review board.

### Sequence of Study Events

First, participants who expressed interest in the study completed a consent appointment via a phone call with the peer counselor. The peer counselor shared many of the same demographic characteristics with the majority of the study participants (eg, race/ethnicity, approximate age, gender, sexual orientation, and geographic location) and had the state of Georgia pre- and posttest HIV counseling certification. During the consent appointment, participants were explained the study procedures, read the consent form, were invited to ask questions, and provided consent via a mobile consenting process where participants typed their name into an electronic consent form that was emailed to them. Upon completing the consent procedures, participants were scheduled for their baseline appointment and were mailed an HIV test to a location of their choice. The arrival of the HIV test kit and the baseline appointment were both scheduled for the same day. The type of HIV test that was mailed to participants was the ORAQuick ADVANCE Rapid HIV-1/2 Antibody Test by OraSure Technolgies, Inc. This test was selected due to its Clinical Laboratory Improvement Amendments waiver, meaning that it is approved for use outside of laboratory settings.

During the baseline appointment, which occurred via video chatting (eg, Skype, FaceTime), participants engaged with the peer counselor in pretest HIV counseling, self-administration of the HIV test (with guidance from the peer counselor), a survey assessment using audio computer-assisted self-interview software (which was completed during the running of the HIV test and took approximately 25 minutes to complete), and posttest HIV counseling. All study procedures, including the self-administration of the HIV test and the baseline survey assessment, were completed during the video chat sessions. The first study appointment took approximately 45 minutes to complete.

The peer-delivered counseling was comprised of content with multiple themes. With the peer counselor's guidance and taking a harm reduction approach, participants worked through a practical and tailored sexual reduction plan based on their reported HIV risk-taking behaviors. Moreover, substance use (ie, alcohol and drug use) in the context of sexual risk taking was assessed and incorporated into the counseling session. Social, emotional, and structural barriers, including anticipated HIV stigma, to engaging in routine HIV testing were evaluated, discussed, and problem solved. In addition, proper referrals to additional services, as needed, were provided by the peer counselor.

The follow-up appointments were completed via a phone call with noncounseling staff members. During the follow-up appointments, the assessment questions and response sets were read to participants and participants provided their answers. Each follow-up appointment took approximately 25 minutes to complete.

### Measures

#### Sociodemographic Characteristics

Participants were asked to report on their age, years of education, employment status, marital status, income level, race/ethnicity, and sexual orientation (ie, whether they identified as same gender loving/gay, bisexual, or heterosexual). Further, participants were asked to report whether they had ever taken an HIV test, and if so, the date of their last HIV test (reported in number of months between date of appointment and date of last test).

#### Feasibility, Acceptability, and HIV Testing Outcomes

Feasibility of recruiting for the study was assessed by tracking the number of men approached, the number who agreed to participate, and the number deemed eligible. Participants’ rates of retention were based on whether they completed the 6-week and the 3-month follow-up sessions. At the end of the study, participants were asked to answer questions regarding their satisfaction with their experience conducting HIV testing via video chatting. Items included: “Would you like to test for HIV at home with video chat-based peer counseling again?” and “Would you recommend testing for HIV at home with video chat-based peer counseling to one of your friends?” Items were dichotomous, 0=no, 1=yes. Also included was “Would you prefer your next HIV test to be in person at an office or at home with peer counseling via video chat?” Response options included “an office” or “at home with video chat-based peer counseling.” Further, participants were asked to rate how satisfied they were with their experience testing at home with video chat–based peer counseling. This item ranged from 0 (not satisfied) to 2 (very satisfied). Additionally, HIV test results, as well as the location of the participants during testing (eg, dorm room, bedroom) were reported.

#### Barriers to HIV Testing

Participants’ barriers to HIV testing—including structural barriers, such as lack of transportation and distance to testing site, as well as concerns regarding confidentiality and fear of testing HIV positive—were assessed using the Barriers to HIV Testing scale [26]. Items included: “The testing site is too far away” and “I am concerned about how I will be treated at the testing site.” Items were rated on a Likert-type scale from 1 (strongly disagree) to 6 (strongly agree).

#### Anticipated HIV Stigma

We assessed the extent to which participants anticipated negative intra- and interpersonal consequences of testing HIV positive in the future using 5 items adapted from the Anticipated HIV Stigma scale [27]. Items included: “I would feel I let myself down if I ever got infected with HIV,” “If I got infected with HIV, no one would date me,” and “I would feel I were not as good a person as others if I got HIV.” All items were rated on a Likert-type scale from 1 (strongly disagree) to 6 (strongly agree).

### Data Analysis

Due to the small sample size, both *t* tests and effect sizes—specifically Cohen *d* [28]—were used to assess treatment effects at the 6-week and 3-month posttest assessments. Cohen *d* was calculated using pre- and posttest scores (at both the 6-week and 3-month assessments) for individual anticipated stigma and barriers to treatment items. This approach was done primarily to understand which barriers to HIV testing, specifically, this modality impacts, and to determine whether anticipated stigma from various sources (eg, family, friends, romantic or sexual partners) are differentially affected by at-home, self-administered HIV testing with video chat–based peer counseling.

In prior research of this nature, Cohen *d* values larger than 0.30 (or −0.30) have been considered to be medium effect sizes and indicate potential change between baseline and follow-ups [29]. Results of significant *t* tests, as well as effect sizes above 0.30 (or below −0.30), are reported for the 6-week and 3-month outcomes in the proceeding text.

## Results

### Sociodemographic Characteristics

Seventy percent (14/20) of men identified as gay, homosexual, or same gender loving, and the remaining 30% (6/20) identified as bisexual. Eighty percent (16/20) identified as Black, non-Hispanic or Latino, 15% (3/20) identified as White, non-Hispanic or Latino, and the remaining individual (1/20, 5%) identified as White, Hispanic or Latino. On average, participants were approximately 28 years of age. Of participants, 100% (20/20) were single, and the majority of participants reported that they were currently working (16/20, 80%). The average length of time since participants’ last HIV test was approximately 12 months (SD=22.9). Of participants, 85% (17/20) had taken at least 1 HIV test in the past (Table 1).

### Feasibility, Acceptability, and HIV Testing Outcomes

Fifty potential participants were screened and described the study opportunity; 15 individuals were screened out based on eligibility criteria, and 7 declined to participate. Twenty-three participants (23/50, 46%) were interested and agreed to participate. Of the 23 men who agreed, 87% (20/23) completed the HIV testing appointment and 90% (18/20) were retained at follow-ups.

Participants’ responses to the satisfaction items demonstrated that all participants found at-home HIV testing with video chat–based peer counseling to be satisfying. All participants reported that they would like to participate in at-home HIV testing with peer counseling via video chat in the future (18/18, 100%) and that they would recommend this modality to one of their friends (18/18, 100%). Further, 72% of participants (13/18) said that they would prefer for their next HIV test to be self-administered at home with counseling from a peer via video chat, as opposed to in a clinic or an office setting (Table 2).

Participants were able to take their HIV tests in a variety of locations, including in their homes (12/20, 60%), in their garages (1/20, 5%), on their porches (1/20, 5%), in dorm rooms (2/20, 10%), at friends’ houses (2/20, 10%), at work (1/20, 5%), and in their car (1/20, 5%). All participants tested HIV negative (20/20, 100%) during the counseling session (Table 3). Further, qualitative observations highlighted that participants frequently reported the importance of being able to administer at-home HIV tests and engage in HIV test counseling via video chat with a peer counselor in a variety of locations. Overwhelmingly, participants reported that they did not want others to know they were taking an HIV test, and that this HIV testing and counseling methodology allowed for greater flexibility in testing location and for control regarding their privacy (ie, who would know that they were testing).

### Barriers to HIV Testing

Participants were less concerned about how they would be treated by people at the testing site at the 6-week posttest (*d*=0.34) than at baseline. Similarly, a number of medium effect sizes emerged at the 3-month follow-up, including less concern regarding (1) lack of transportation (*d*=0.31), (2) testing sites being too far away (*d*=0.32), (3) how they will be treated at the testing site (*d*=0.37), and (4) finding out the results of their HIV tests (*d*=0.37). For each of these items, participants’ barriers were reduced from baseline to the 3-month posttest (Table 4). A second qualitative observation included noting that participants reported the video chat–based peer counseling component of their HIV testing experiences as an important and helpful opportunity to troubleshoot barriers to HIV testing in the future.

**Table 1:** Demographic characteristics of men who have sex with men (MSM) recruited from the Atlanta, GA, area for HIV testing via video chat.

Demographic information		Mean (range) or n	SD or %
Age, mean (range), SD		28.05 (20-44)	6.80
Education, mean (range), SD		2.15 (1-4)	1.23
Income, mean (range), SD		3.20 (1-7)	1.80
**Sexual orientation, n, %**			
	Gay/homosexual/same gender loving	14	70
	Bisexual	6	30
**Race/ethnicity, n, %**			
	Black, non-Hispanic or Latino	16	80
	White, non-Hispanic or Latino	3	15
	White, Hispanic or Latino	1	5
**Gender, n, %**			
	Male	19	95
	Transgender female	1	5
**Marital status, n, %**			
	Single	20	100
	Domestic partnership/Civil union	0	0
**Employment status, n, %**			
	Unemployed	4	20
	Working	16	80
Time since last HIV test in months, mean (range), SD		12.31 (1.07-95.40)	22.93
**Have you ever taken an HIV test in the past? n, %**			
	Yes	17	85
	No	3	15.0

**Table 2:** Participants’ satisfaction with at-home HIV testing with peer counseling via video chat at the 3-month follow-up.

Patient satisfaction questions		Mean (range) or n	SD or %
How satisfied were you with at-home HIV testing with video chat-based peer counseling? mean (range), SD		1.89 (0-2)	0.32
**Would you like to test for HIV at home with video chat–based peer counseling again? mean (range), SD**			
	Yes	18	100
	No	0	0
**Would you recommend testing for HIV at home with video chat–based peer counseling to one of your friends? mean (range), SD**			
	Yes	18	100
	No	0	0
**Would you prefer your next HIV test to be in person at an office or at home with video chat–based peer counseling? mean (range), SD**			
	At an office	5	28
	At home with video chat-based peer counseling	13	72

**Table 3:** Participants’ HIV testing outcomes from at-home HIV testing with HIV counseling via video chat.

Testing outcomes		n (%)
**Result of HIV test**		
	HIV negative	20 (100)
	HIV positive	0 (0)
**Location of HIV test**		
	Home, general	12 (60)
	Home, in garage	1 (5)
	Home, on porch	1 (5)
	In dorm room	2 (10)
	At a friend’s house	2 (10)
	Inside of car	1 (5)
	At work	1 (5)

**Table 4:** Means and standard deviations of the anticipated stigma and barriers to testing items at baseline, 6-week, and 3-month follow-ups.

Variable		Baseline		6-week post		3-month post		6-week effect size	3-month effect size	*P* value
		M	SD	M	SD	M	SD			
**Barriers to testing items**														
	I don’t have transportation to site.	1.61	1.50	1.56	1.34	1.22	0.94	0.04	0.31	.13
	I don’t have enough time.	1.94	1.80	1.78	1.35	1.67	1.41	0.10	0.17	.57
	The testing site is too far away.	1.72	1.71	1.50	1.04	1.28	0.96	0.16	0.32	.15
	I don’t know where to go for testing.	1.39	1.24	1.33	0.97	1.72	1.67	0.05	−0.22	.39
	I am concerned about how I will be treated by people at the testing site.	2.50	2.07	1.89	1.53	1.83	1.52	0.34	0.37	.19
	I have had a bad HIV testing experience in the past.	1.72	1.64	1.39	1.15	1.61	1.50	0.23	0.07	.76
	I can’t afford treatment, so why get tested?	1.39	1.24	1.33	1.03	1.33	1.03	0.05	0.05	.75
	I don’t want to know the results.	2.11	2.03	1.72	1.57	1.50	1.20	0.21	0.37	.15
	People might recognize me at the testing site.	2.39	2.20	2.89	2.08	2.00	1.72	−0.23	0.20	.42
	I am worried about my health information being kept confidential.	2.00	1.97	2.50	1.86	1.72	1.23	−0.26	0.17	.58
**Anticipated stigma items**										
	I would let myself down if I ever got infected with HIV.	5.17	1.30	5.56	0.71	5.22	1.31	−0.37	−0.04	.90
	I would let my family and friends down if I ever got infected with HIV.	3.78	1.99	4.50	1.82	4.67	1.88	−0.38	−0.46	.04
	If I got infected with HIV, no one would date me.	2.17	1.69	2.33	1.68	2.33	1.94	−0.09	−0.09	.66
	If I got infected with HIV, men would not want to have sex with me.	3.11	1.97	2.33	1.50	2.28	1.81	0.44	0.44	.07
	I would feel I were not as good a person if I got HIV.	2.28	1.71	2.78	1.93	2.33	1.78	−0.27	−0.03	.90

### Anticipated HIV Stigma

An effect size of *d*=−0.37 for the “I would let myself down if I ever got infected with HIV” item was noted, indicating that, at the 6-week follow-up, participants were more likely to feel that they would let themselves down if they ever got infected with HIV. Moreover, for the item “I would let my family and friends down if I ever got infected with HIV,” *d*=−0.38 at the 6-week follow-up and *d*=−0.46 at the 3-month follow-up, suggesting that participants were more likely to feel that they would let their family and friends down if they got infected with HIV at follow-ups than baseline. Further, there was a significantly different change in mean scores from pre- to 3-month posttest for this item, *t*_(17)_=.16, *P*=.04. For the item “If I got infected with HIV, men would not want to have sex with me,” *d*=0.44 at both the 6-week and 3-month follow-ups, meaning that participants were less likely to feel that getting infected with HIV would result in men not wanting to have sex with them at the follow-ups than at baseline (Table 4).

## Discussion

### Principal Findings

The results of this test of concept study suggest that at-home, self-administered HIV testing with peer counseling via video chat is well received and has the potential to benefit participants and to serve as a viable and novel HIV testing and counseling approach. A substantial number of the men who were asked to participate in this study did so and those who did participate found this new form of HIV testing and counseling to be a highly satisfying experience. Further, a majority of participants reported preferring this approach to testing over testing in an office setting in the future. All participants reported that they would like to test for HIV using this method again (ie, at home testing with peer counseling via video chat) and that they would recommend to their friends this method of HIV testing and counseling. These findings suggest that this HIV testing and counseling approach is well-received by MSM, which points to its potential to make an impact on improving HIV testing uptake and frequency among this higher risk population [30]. These findings warrant support for further study of at-home HIV testing and peer counseling via video chat.

The benefits of administering Web-based HIV counseling are numerous, including being less time-intensive and more convenient, as well as the relative anonymity for participants and the flexibility with which they can choose the location to take their tests [7,31]. Some participants in this study tested in places other than their homes, including in their cars, at their workplaces, in their garages, and at their friends’ homes. However, it is important to note that all participants were encouraged to find a safe and comfortable place to test where their privacy could be protected. In cases where participants lived with relatives or friends, the ability to take their tests outside of the home was beneficial for maintaining their privacy and the Web-based, video chatting format of the counseling did well to allow for this important flexibility.

Interestingly, a number of moderate effect sizes were found, including a notable decrease in barriers to testing, namely logistical (eg, transportation) and psychosocial (eg, fear of discrimination or being recognized at the testing site) barriers [32-34]. The present study is a pilot study with a small sample size, and, as such, these results should be interpreted with caution; however, they provide preliminary support for findings that could be further evaluated by larger scale studies. In previous research, these barriers have been identified as places of weakness in the HIV continuum of care, particularly for the most vulnerable communities (eg, BMSM), for whom the burden of undetected HIV infection strongly persists [2,35,36]. Arguably, these barriers may have been lessened because of the Web-based nature of this study. It is possible that by introducing participants to an alternative route to HIV testing (ie, testing at home with video chat–based peer counseling) previously held logistical or stigma-related concerns about accessing HIV testing were abated. Further, the peer counselors worked to problem solve barriers to seeking out local HIV testing sites during the counseling sessions.

This study found that anticipated HIV stigma both increased and decreased from pre- to posttest. While participants were more likely to feel that they would let their family and friends down if they were to be infected with HIV in the future, they were less likely to report that men would not want to have sex with them if they became infected with HIV.  It is possible that exposure to new HIV knowledge or having conversations with a peer educator about HIV stimulated newfound anxiety among the participants regarding their HIV vulnerability and/or having to manage telling family and friends about an HIV diagnosis.  As part of the sexual risk reduction portion of the counseling experience, however, participants conversed with the peer educator about partner selection strategies, which may have led to the decreased scores related to HIV stigma from romantic or sexual partners at the 6-week and 3-month posttests.  This decrease in anticipated HIV stigma is important because research has indicated that HIV stigma negatively impacts the health of MSM by limiting access to health care, discouraging routine HIV testing, and contributing to stress, social isolation, and risky sex behaviors [37-40]. Future testing modalities of this nature should include discussions concerning not only sexual and romantic partners, but family and friends as well [41].

### Strengths and Limitations

To our knowledge, this is the first test of concept of at-home HIV testing with video chat–based peer counseling for MSM. This study adds to the extant literature by identifying the feasibility of HIV test counseling via video chat. One strength of the current study’s sample is that the majority of participants identified as African-American or Black. The need for novel HIV testing strategies for BMSM is urgent given the alarmingly high rates of HIV among this community, and the presently assessed modality—at-home, self-administered HIV testing with peer counseling via video chat—was well-received by BMSM. However, it is also worth noting that the study used surveys, which relied on self-reporting of potentially sensitive experiences and behaviors and, therefore, may be prone to bias. Additionally, social desirability bias may have impacted the results of the study by affecting study satisfaction–related outcomes, among other study data points. Further, participants were asked whether they wanted their next HIV test to be at an office or at home with video chat–based peer counseling, but did not provide participants with other testing venue options to choose from (eg, community clinic). Another important limitation to the study is the cost associated with at-home HIV test kits. Given the expense of personally purchased, at-home HIV test kits, community-based organizations must provide test kits for uptake of the proposed testing strategy.

Protocols were developed and put in place to support participants testing HIV positive during the HIV testing piece of the video chat–based peer counseling. Specifically, our procedures for providing participants who test HIV positive appropriate support and linkage to care included the following multiple steps: (1) the provision of Center for Disease Control–based post-HIV test counseling for individuals testing HIV positive; (2) evaluation of mental health status and referral to immediate or delayed care as determined by the counselor; (3) linkage to long-term HIV care organization of the counselor’s referral and/or the participant’s choice; (4) the arrangement of initial HIV care appointment by the counselor; (5) review with participant, as needed, services provided by selected HIV care organization (eg, health insurance assistance, substance use and mental health treatment, housing, transportation, support groups, medication counseling, transportation, etc); (6) re-contacting the participant 2 days, 2 weeks, and 2 months posttesting appointment to ensure proper follow-up care and to problem solve obstacles; and (7) report test results to the State Health Department (additional details on at-home HIV testing protocol can be obtained by contacting the primary author of this manuscript). Though we had these procedures in place, all participants in our sample tested HIV negative. It is possible that the study results were impacted by this particular outcome. Future studies are needed to assess the successes and challenges of linking individuals who test HIV positive to care.

### Conclusions

By assessing the acceptance and feasibility of at-home, self-administered HIV testing with video chat–based peer counseling among this population, this study gains a key insight as to how such an intervention can be best delivered to individuals in need of HIV testing and counseling, but who may not have access to or who may not prefer more common routes to testing and other services, including clinic-based services. The study demonstrated feasibility, and the participants were satisfied with their experiences, indicating not only that this testing and counseling modality could likely be replicated, but also that a Web-based, video chat approach to HIV counseling is acceptable to participants. Given the paucity of alternative venue HIV testing locations available to MSM, as well as the number of MSM who are not able or willing to access services, this study is an important step toward filling an unmet need.

## Abbreviations

BMSM: men who have sex with men who identify as African-American or Black
HIV: human immunodeficiency virus
MSM: men who have sex with men
STI(s): sexually transmitted infection(s)
